# Health-related quality of life outcomes of surgery for diffuse glioma: A systematic review and pooled analysis

**DOI:** 10.1093/nop/npaf111

**Published:** 2025-10-25

**Authors:** Yash Akkara, Ryan Afreen, Raymund L Yong

**Affiliations:** Imperial College London School of Medicine, London (Y.A.); Icahn School of Medicine at Mount Sinai, New York (R.A.); Department of Neurosurgery, Icahn School of Medicine at Mount Sinai, New York (R.L.Y.)

**Keywords:** diffuse glioma, glioblastoma, health-related quality of life, surgery

## Abstract

**Background:**

Although progress has been made in understanding the effects of adjuvant therapy on health-related quality of life (HR-QoL) in diffuse glioma patients, less is known about the impact of surgical resection. To address this, we conducted a systematic review and pooled quantitative analysis.

**Methods:**

PubMed, MEDLINE, and Embase were searched for studies measuring HR-QoL before and after surgery for WHO grade 2-4 adult-type diffuse gliomas. Inclusion was limited to prospective cohort studies and trials on adults with ≥1 month of postoperative follow-up. Metric outcomes were assessed with pooled odds, competing risk analysis, and meta-regression using a random effects model. Bias was assessed using the Newcastle-Ottawa Scale and Cochrane Risk of Bias 2.0 tool.

**Results:**

Twelve studies comprising 1000 patients were included. The pooled odds of an unfavorable versus favorable HR-QoL change compared to baseline was not significantly different from 1 within 3 months of surgery (0.843, 95% CI, 0.339-2.100), but significantly less than 1 at final follow-up (0.481, 95% CI, 0.260-0.888). The cumulative incidence of favorable HR-QoL change was significantly higher than that of unfavorable change, with the incidence curves separating after 3 months (χ^2^(1) = 95.0, *P* < .001). This was attributable to EQ-5D and EORTC QLQ-C30 but not SF-36. Studies with younger patients, more high-grade tumors, and lower gross total resection rates showed worse outcomes.

**Conclusion:**

Surgical resection can maintain or improve HR-QoL, but patients at risk of deterioration should be identified early. Future studies must carefully select and interpret HR-QoL instruments, as preference-based and non-preference-based tools may lack comparability.

Key Points

- Surgery improves or stabilizes HR-QoL beyond 3 months in diffuse glioma patients.
- Younger age, HGG, and low GTR predict worse long-term HR-QoL outcomes.
- HR-QoL instrument choice impacts results; metrics vary in sensitivity and focus.

Importance of the Study

This study is the first to quantitatively pool HR-QoL outcomes following surgical resection in adults with diffuse gliomas, offering a clearer understanding of how surgery impacts patient well-being over time. Unlike previous analyses focused on adjuvant therapy, our study isolates the effect of surgery, showing that HR-QoL generally stabilizes or improves beyond 3 months postoperatively. By identifying key predictors of long-term HR-QoL decline, such as younger age, high-grade gliomas, and lower resection rates, this work highlights high-risk groups that may benefit from targeted interventions. Additionally, our findings underscore the influence of HR-QoL measurement tools on study outcomes, emphasizing the need for thoughtful selection of instruments in future research. These insights will aid clinicians in balancing surgical aggressiveness with quality-of-life considerations and support more nuanced, patient-centered care planning in diffuse glioma management.

Adult-type diffuse gliomas are associated with significant morbidity and mortality. Low-grade gliomas (LGGs, WHO grade 2), although relatively uncommon among central nervous system tumors, often present with neurocognitive deficits such as memory impairment, attention difficulties, and executive dysfunction, and tend to occur in younger patients with longer survival prospects.^1^^,^^2^ In contrast, high-grade gliomas (HGGs, WHO grades 3-4), including glioblastoma (GBM), tend to occur in older patients and are more frequently associated with symptoms of mass effect and edema at presentation, as well as steeper declines in neurocognitive function during disease progression.^3^

When safely possible, maximal surgical resection is considered the first-line treatment for gliomas, with the goals of reducing tumor burden, improving symptoms, and confirming the molecular-histological diagnosis.^4^ For HGGs and high-risk LGGs, surgical therapy is followed by adjuvant temozolomide (TMZ)-based chemoradiation, which has been shown to improve overall survival.^5^ However, no curative therapy currently exists. Due to factors such as treatment-resistant tumor cells, the infiltration of malignant cells into distant regions of the brain, and insufficient penetrance of systemically administered therapies, disease progression remains inevitable. Studies have also highlighted the possible lack of survival benefit from surgical resection or other tumor-specific therapy in elderly patients, particularly with MGMT-unmethylated GBMs.^6^^,^^7^

In light of this, optimization of health-related quality of life (HR-QoL) should be considered a primary goal when undertaking any course of oncologic treatment.^8^^,^^9^ HR-QoL is defined as a patient’s perception of their physical and occupational function, physiological state, social interactions, and somatic sensations, as influenced by their medical condition and/or consequences of therapy.^10^ Generic HR-QoL metrics assess broad aspects of health status and allow comparisons across diseases, whereas disease-specific tools are tailored to capture symptoms and functional domains most relevant to a particular condition. Additionally, preference-based measures, such as the EQ-5D, provide utility scores that can be used in health economic evaluations,^11^ while non-preference-based tools, such as the EORTC QLQ-C30 or SF-36, yield detailed profiles of functioning and symptoms without generating utility values.^12^^,^^13^ Studies have highlighted the strong correlations between HR-QoL and personal health, cognitive function, and employment.^14^^,^^15^ Although the neurofunctional outcomes of glioma surgery have been frequently studied within the literature,^16^ few studies have directly explored the effect of surgical resection on HR-QoL domains such as well-being, comfort, and health satisfaction. To more fully assess HR-QoL trends and their predictors during the perioperative period and beyond, we aimed to conduct a systematic review and pooled analysis of all studies to date that provide matched pre- and postoperative longitudinal HR-QoL data on glioma patients using 1 or more validated instruments.

## Methods

### Structured Search

A structured search was conducted in accordance with guidelines outlined by the Preferred Reporting Items for Systematic Review and Meta-Analysis (PRISMA) checklist.^17^ Databases searched included PubMed, MEDLINE, and Embase from inception to December 2024. The keywords “(Glioma OR Glioblastoma OR GBM OR Astrocytoma OR Oligodendroglioma OR Oligoastrocytoma) AND (Surgery OR Operative OR Resection OR Operation) AND (Quality of Life OR QOL OR Quality Outcomes OR Health-related quality of life OR HR-QoL)” were used in MeSH and text-word formats.

### Inclusion and Exclusion Criteria

Inclusion was restricted to clinical trials and prospective cohort studies in English. Studies were required to have a sample size of ≥10 participants, all of whom were ≥16 years of age. Participants were limited to patients with a diagnosis of an adult-type diffuse glioma of grade 2 or above according to the 2021 WHO classification of CNS tumors.^18^ Included studies were required to measure HR-QoL using any validated instrument at a minimum of 2 time points, with the initial measurement at baseline before surgical intervention, such that it was possible to determine the number of patients experiencing clinically significant changes at each time point. Following surgery, studies were required to report any additional therapy given to patients. While studies that included patients with recurrent gliomas were included, it was required that the study provided HR-QoL measurements prior to the patients’ most recent glioma surgery. A minimum follow-up time of 1 month after surgery was required.

### Data Extraction and Bias Assessment

Studies from the database search were input into the systematic review software Covidence (covidence.org). Three reviewers independently completed initial abstract and title screening, followed by full-text screening in accordance with the inclusion criteria. Conflicts in decisions were discussed until a consensus was reached with the assistance of the senior author. The following study characteristics and patient variables were extracted: author, study name, study design, country, sample size (at baseline and follow-ups), sex, population age (mean, median, range), Karnofsky Performance Status (KPS, at baseline and last follow-up), reported symptoms, tumor characteristics (histology, grade, lobe, location, and IDH status), surgical factors (complete excision rate, median excision rate, complications), treatment factors (neoadjuvant/adjuvant therapy, management outside standard of care), HR-QoL (metrics, measurements at baseline and follow-up), overall follow-up, and survival (overall, mean, and/or median). Gross total resection was defined as the absence of contrast-enhancing residual lesion in HGGs and the absence of bulky T2-hyperintense residual tumor in LGGs.

Quality assessment was conducted independently by all reviewers, with any conflicts discussed with the senior author until consensus was reached. The Newcastle-Ottawa Scale (NOS) was used for observational studies. The Cochrane Risk of Bias 2.0 tool was used for interventional studies.

### Statistical Analysis

Odds were calculated using tallies of the number of patients reporting unfavorable (defined as worsened) or favorable (defined as stable or improved) HR-QoL outcomes in each study at each time point. Because scores from preference-based and non-preference-based HR-QoL metrics cannot be directly compared to each other, we used the minimal clinically important difference (MCID) for each instrument, as defined in each study, to dichotomize HR-QoL changes from the patient’s preoperative baseline as either unfavorable or favorable, enabling analyses across heterogeneous measures to be pooled. In general, this was a 0.15 change in index for the EQ-5D-3L, a 10-point change for the EORTC QLQ-C30, and a 1 SD change from the mean population physical component or mental component score for the SF-36. Study time points were pre-defined as up to 3 months following surgery (T1) and at the last follow-up after 3 months (T2). Studies were grouped by the primary HR-QoL metric employed, and a random-effects model was used to assign weight to each study based on within-group and between-group variance. Studies with more than 1 HR-QoL metric or treatment arm were split into their respective cohorts, with the HR-QoL changes of each cohort considered separately. Cumulative incidence functions of patients experiencing favorable or unfavorable HR-QoL change compared to their preoperative baseline were compared using Gray’s test.^19^ Simple and multiple linear meta-regression was performed using the ordinary least squares method without weighting of the dependent variable. Backwards stepwise optimization was used for all variables found to be significant in the univariate analysis, with the multiple-linear regression iterated until the Akaike Information Criterion value was minimized or all variables reported *P* < .1. The normality of residuals was assessed using the D’Agostino-Pearson test. Data were compiled and analyzed using Prism 10.1.1 (GraphPad Software) and Rstudio 2025.05.1 (Posit Software, PBC) using the tidycmprsk package.

### Study Registration

This review has been registered with the international prospective register of systematic reviews (PROSPERO). Registration ID: CRD42024557239.

## Results

### Search and Patient Characteristics

**Figure 1.**
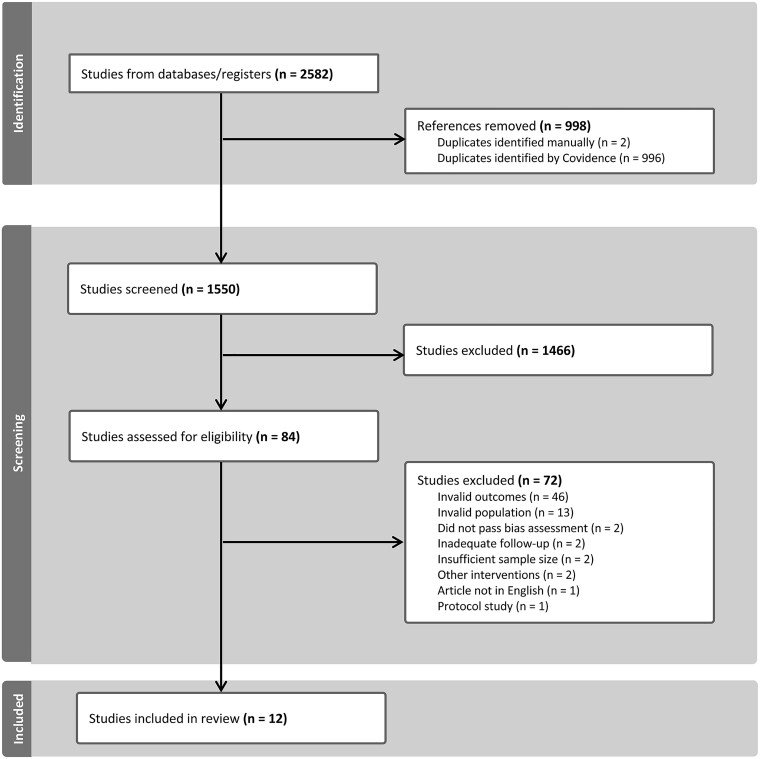
PRISMA diagram of identified, screened, and included studies.

We identified 2548 studies during the initial search, of which 998 were duplicates (Figure 1). The remaining 1550 studies underwent title and abstract screening, resulting in 1466 studies excluded. Eighty-four studies underwent full-text screening, of which 72 were excluded. The most common reasons for exclusion included a lack of a preoperative HR-QoL measurement, patient populations outside the inclusion criteria, and a lack of sufficient follow-up. This left 12 studies for the final pooled analysis, including 11 prospective cohort studies and 1 randomized controlled trial (Table 1).^20–31^ The final pooled sample comprised 1000 participants (606 male, 394 female), with a mean age of 55.0 years (Table 2). There were 175 (17.5%), 118 (11.8%), and 707 (70.7%) patients with grades 2-4 gliomas, respectively. Most patients underwent primary operations (84.8%), while the remaining patients underwent re-operations for recurrent or progressive gliomas. The mean follow-up time across all studies was 5.1 months.

**Table 1.** Characteristics of included studies

Study	Instrument used	Design	Baseline sample size	Reassessment time points	MCID used
Jakola et al. (2011)^20^	EQ-5D	Single cohort	61	Baseline, 1 month	0.15 change in index score
Jakola et al. (2011)^21^	EQ-5D	Single cohort	88	Baseline, 1.5 months	0.15 change in index score
Jakola et al. (2015)^22^	EQ-5D	Single cohort	22	Baseline, 1 month	0.15 change in index score
Sagberg et al. (2016)^23^	EQ-5D	Single cohort	30	Baseline, 4, 6, 10, 12 months	0.15 change in index score
Wolf et al. (2016)^24^	EORTC QLQ-C30, QLQ-BN20	Single cohort	22	Baseline, 3 months	Reliable change index >1.96
Jakola et al. (2017)^25^	EQ-5D	Single cohort	73	Baseline, 6 months	0.15 change in index score
Drewes et al. (2018)^26^	EQ-5D	Dual cohort (LGG and HGG)	136	Baseline, 1, 6 months	0.15 change in index score
Sagberg et al. (2019)^27^	EQ-5D	Single cohort	112	Baseline, 3 months	0.15 change in index score
Liu et al. (2020)^28^	EORTC QLQ-C30	RCT (control vs. ERAS)	65	Baseline, 3, 6 months	10 points
Leonetti et al. (2021)^29^	SF-36	Dual cohort (LGG and HGG)	145	Baseline, 1, 3, 6, 12 months	1 SD from the mean PCS or MCS score
Taskiran et al. (2021)^30^	EORTC QLQ-C30	Single cohort	49	Baseline, 1 month	10 points
Rubin et al. (2022)^31^	EQ-5D	Dual cohort (primary and recurrent)	197	Baseline 3 months	0.15 change in index score

Abbreviations: ERAS, enhanced recovery after surgery protocol; HGG, high-grade diffuse glioma; LGG, low-grade diffuse glioma; MCS, mental component scale; PCS, physical component scale; RCT, randomized controlled trial.

**Table 2.** Characteristics of pooled patient cohort

Variable		Value
Sex, *n* (%)	Male	606 (60.6%)
	Female	394 (39.4%)
Mean age (years)		55.0
Age range (years)		18-86
Surgical History, *n* (%)	Primary	848 (84.8%)
	Reoperation	152 (15.2%)
Median KPS (range)	Baseline	75 (15)
	Last follow-up	75 (15)
Grade, *n* (%)	Grade 2	175 (17.5%)
	Grade 3	118 (11.8%)
	Grade 4	707 (70.7%)
Histology, *n* (%)	Astrocytoma	120 (12.0%)
	Oligodendroglioma	55 (5.5%)
	Anaplastic astrocytoma	113 (11.3%)
	Anaplastic oligodendroglioma	5 (0.5%)
	Glioblastoma	707 (70.7)
Hemisphere, *n* (%)	Right	495 (49.5%)
	Left	505 (50.5%)
Lobe, *n* (%)	Frontal	151 (15.1%)
	Temporal	115 (11.5%)
	Parietal	63 (6.3%)
	Insular	40 (4.0%)
	Midline structures	17 (1.7%)
	Occipital	8 (0.8%)
	Not reported	606 (60.6%)

### Risk of Bias Assessment

Six studies scored well (7-8) for bias in the NOS, while 5 scored moderately (5-6) (Table S1). The study assessed using the Cochrane Risk of Bias 2.0 tool scored at a low level of bias. We tested for correlations between study sample size and the reported odds of worsened HR-QoL, both at T1 (*r* = 0.0003, 95% CI, −0.0014 to 0.0020) and at T2 (*r* = −0.0004, 95% CI, −0.0019 to 0.0012), and found no evidence that odds values differed between smaller and larger studies in a systematic manner that would indicate publication bias.

### Surgical Outcomes

The overall rate of gross total resection was 52.2%. Following surgery, 73.5% of patients received 60 Gy of radiation therapy concomitantly with TMZ as per the Stupp protocol. Using the Landriel-Ibanez classification,^32^ 157 (15.7%), 65 (6.5%), 21 (2.1%), and 6 (0.6%) patients experienced grades 1-4 complications, respectively. The most common complications were new neurological deficits, hematoma, and surgical site infections.

### HR-QoL Outcomes

**Figure 2.**
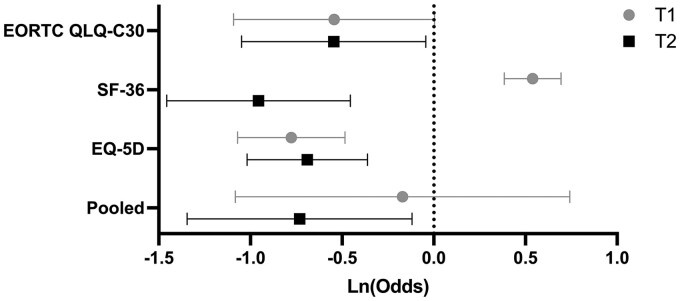
Forest plot of odds of an unfavorable versus favorable HR-QoL change following glioma surgery within 3 months postoperatively (T1) and at last follow-up (T2).

At T1, the pooled mean odds of a worsened versus favorable (defined as stable or improved) change in HR-QoL compared to the preoperative baseline across all studies were 0.843 (95% CI, 0.339-2.100), which was not significantly different than 1:1 odds, suggesting no significant HR-QoL changes versus baseline. However, at the last follow-up time point (T2), the mean pooled odds were significantly less than 1 at 0.481 (95% CI, 0.260-0.888), indicating that surviving patients were on average more likely to report stable or improved, rather than worsened, HR-QoL compared to their preoperative baseline at later time points (3-12 months) following surgery (Figure 2).

Across studies, the EQ-5D-3L was the most employed primary metric (*n* = 8), followed by the EORTC QLQ-C30 (*n* = 3) and SF-36 (*n* = 1). Considering that studies may have employed more than 1 metric on the same patient, the EQ-5D-3L, EORTC QLQ-C30, and SF-36 were used at T1 on 616, 136, and 290 patients, respectively, and 719, 136, and 290 patients at T2, respectively. At T1, the pooled odds of a worsened versus a favorable outcome in studies using the EORTC QLQ-C30 (0.58, 95% CI, 0.34-1.00, *P* = .051) and EQ-5D-3L (0.46, 95% CI, 0.34-0.62, *P* < .0001) were significantly less than 1. In contrast, the pooled odds of worsening were significantly greater than 1 in studies employing the SF-36 (1.71, 95% CI, 1.47-2.00, *P* < 0.0001). At T2, the EORTC QLQ-C30 (0.58, 95% CI, 0.35-0.96, *P* = .033), EQ-5D-3L (0.50, 95% CI, 0.36-0.70, *P* < 0.0001), and SF-36 (0.38, 95% CI, 0.23-0.63, *P* < .001) all reported significantly less than 1 odds of worsened HR-QoL compared to baseline (Figure 2), indicating HR-QoL stability and improvement.

**Figure 3.**
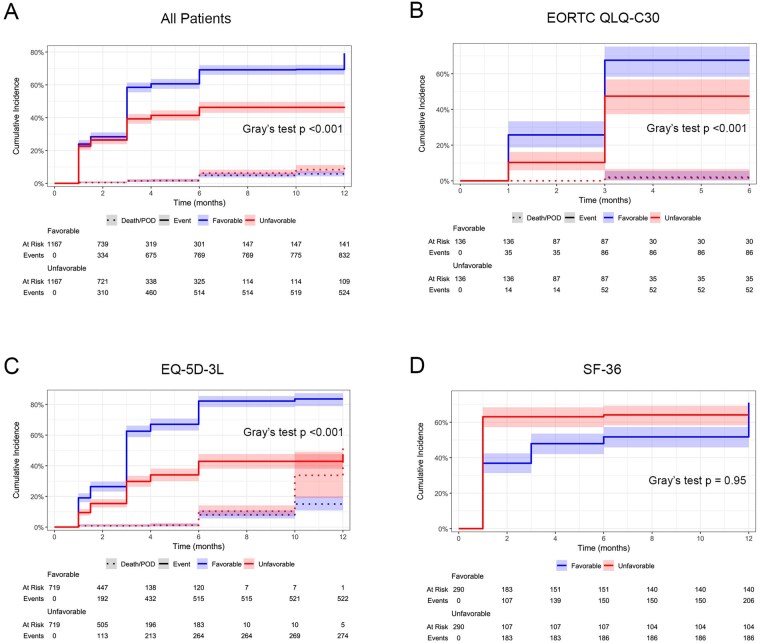
Cumulative incidence functions of favorable (improved or stable) and unfavorable HR-QoL change compared to preoperative baseline with death or progression of disease (POD) as a competing risk. (A) The full pooled cohort; (B) studies employing the EORTC QLQ-C30; (C) studies employing the EQ-5D-3L; and (D) studies employing the SF-36.

To account for patients who did not complete HR-QoL questionnaires at all study time points, we performed a pooled competing risk analysis in which the reporting of favorable (improved/stable) and unfavorable (worsened) HR-QoL compared to the baseline were events of interest, and failure to provide HR-QoL data due to symptom progression or death was a competing risk. Patients who had not died but were lost to follow-up for unknown reasons were censored. Pooling outcomes for all HR-QoL metrics, the cumulative incidence of a favorable event was significantly higher than that of an unfavorable event during the first year after surgery, with the incidence curves separating after 3 months (Figure 3A) (χ^2^(1) = 95.0, *P* < .001). HR-QoL metrics were then analyzed individually. Patients assessed using the EORTC QLQ-C30 (χ^2^(1) = 13.8, *P* < .001) (Figure 3B) and EQ-5D-3L (χ^2^(1) = 161, *P* < .001) (Figure 3C) similarly exhibited a significantly higher cumulative incidence of a favorable versus unfavorable HR-QoL outcome compared to baseline, while a significant difference was not observed for patients assessed using the SF-36 (χ^2^(1) = 0.004, *P* = .95) (Figure 3D). In the EQ-5D-3L subgroup, the cumulative incidence of death or symptom progression preventing HR-QoL reporting was higher for patients who experienced unfavorable versus favorable changes in HR-QoL compared to baseline (χ^2^(1) = 7.14, *P* = .008), with this becoming most apparent after 10 months postoperatively. No difference was seen in the other subgroups or in the pooled cohort.

### Univariate and Multivariate Meta-Regression

We performed simple and multiple linear meta-regressions (Table 3), reporting the odds of worsened HR-QoL as a function of several study sample characteristics, both at T1 and T2. In the univariate analysis, both lower mean age of the study population and higher rate of GTR were significantly associated with worsened HR-QoL compared to baseline at T1, while at T2, a higher proportion of HGGs and a higher rate of adjuvant therapy were associated with worsened HR-QoL compared to baseline. Using these 4 variables in the multivariate analysis, we found that mean age of the study population was an independent predictor of the odds of worsened HR-QoL compared to baseline at T1 (β = −0.0767, *P* = .0054), while mean age (β = −0.2183, *P* = 0.0377), proportion of HGGs (β = 0.0331, *P* = .0178), and rate of GTR (β = −0.0426, *P* = .0345) were all independent predictors at T2.

**Table 3.** Simple linear and multiple meta-regression of ln(odds) of significantly worsened HR-QoL postoperatively as a function of various study population characteristics

Study characteristic	T1			T2		
	β	95% CI	*P*-value	β	95% CI	*P*-value
**Univariate analysis**						
Proportion male	0.00578	−0.00372 to 0.0153	0.2154	0.00458	−0.00925 to 0.0101	0.9290
Mean age	−0.0374	−0.0689 to −0.00575	**0.0238**	0.0271	−0.00713 to 0.0614	0.1121
% Left hemisphere tumors	0.0288	−0.0161 to 0.0738	0.1889	−0.00480	−0.0432 to 0.0336	0.7922
% Deep/midline/insular tumors	−0.00819	−0.0329 to 0.0165	0.4893	0.00792	−0.0129 to 0.0287	0.4300
% KPS <70	−0.00760	−0.0223 to 0.00712	0.2827	−0.00511	−0.0199 to 0.00969	0.4692
% High-grade glioma	−0.00238	−0.00986 to 0.00510	0.5098	0.00712	0.000380 to 0.0139	**0.0396**
Rate of GTR	0.0121	0.00362 to 0.0207	**0.0081**	−0.00192	−0.0128 to 0.00891	0.7114
Surgical complication rate	0.0161	−0.0119 to 0.0441	0.2249	0.00729	−0.0270 to 0.0416	0.6416
% Receiving adjuvant therapy	0.00138	−0.0141 to 0.0169	0.8507	0.0136	3.649 × 10^−5^ to 0.02717	**0.0495**
**Multivariate analysis**						
Mean age	−0.0767	−0.126 to −0.0269	**0.0054**	−0.218	−0.421 to −0.0155	**0.0377**
% High grade glioma	0.0095	−0.000342 to 0.0194	0.0573	0.0331	0.00721 to 0.0590	**0.0178**
Rate of GTR	–		–	−0.0426	−0.0813 to −0.00387	**0.0345**

Abbreviations: GTR, gross total resection; T1, up to 3 months postoperatively; T2, at last follow-up.

## Discussion

This study, to our knowledge, is the first to perform a pooled quantitative analysis of changes in HR-QoL scores following surgery for adult-type diffuse gliomas. Our review identified 12 studies examining HR-QoL outcomes of patients with gliomas at baseline and following surgical resection. The pooled odds and competing risk analyses strongly suggest that surgical resection provides meaningful improvements in HR-QoL in patients with gliomas, particularly at longer follow-up intervals up to 1 year postoperatively. Generally, patients undergoing surgery seem to maintain HR-QoL levels compared to their baseline initially, with no significant difference in the proportion of patients improving, remaining stable, or worsening for 3 months. Beyond that, patients were more likely to report favorable versus unfavorable HR-QoL change, suggesting that resection appears to be effective at stabilizing symptoms that impact HR-QoL until the adjuvant therapy phase is entered.

In the only other published pooled analysis of HR-QoL data from diffuse glioma patients we were able to identify, Coomans et al.^33^ reported on 5539 patients, who participated in 15 clinical trials spanning 2006-2018. Unlike our study, however, changes in HR-QoL (as measured by the EORTC QLQ-C30 and QLQ-BN20) were tracked from the time of randomization to experimental or standard adjuvant therapy. Because randomization occurred only after a tissue diagnosis had been made with resection or biopsy, these trials were excluded from our analysis. Deterioration from this postoperative baseline by a clinically meaningful amount without subsequent improvement occurred in 47% of patients overall during the disease progression-free period, which is very similar to our finding of a 46.7% cumulative incidence of patients experiencing worsened HR-QoL compared to their *preoperative* baseline at 12 months in our competing risk analysis. This further supports the notion that significant declines in HR-QoL experienced by diffuse glioma patients are more likely to be attributable to the effects of adjuvant therapy and tumor progression than to factors specifically associated with surgery.

Our subgroup analyses by metric demonstrated differences in pooled odds among patients assessed with the EQ-5D-3L, EORTC QLQ-C30, or SF-36 at time points up to 3 months. The odds of a worsened EQ-5D-3L score were significantly less than 1 (indicating HR-QoL stability or improvement), whereas for the EORTC QLQ-C30 and SF-36, these odds were marginally less than 1 and significantly greater than 1 (suggesting HR-QoL worsening), respectively. This heterogeneity among the HR-QoL instruments following surgery may suggest that the follow-up window of 3 months may be insufficient for capturing HR-QoL trends across the full disease course of diffuse gliomas, potentially due to patients still recovering from surgery and experiencing varying effects during the initiation of adjuvant therapy. At time points after 3 months, however, all metrics concordantly showed that HR-QoL in surviving patients was more likely to be equal to or greater than baseline than below. The mean follow-up of included studies was approximately 5 months, with 6 studies reporting follow-up data beyond 6 months. As such, these findings emphasize the need for longer data collection windows when designing studies that examine HR-QoL in glioma patients.

Within subgroup competing risk analyses by metric, both the EQ-5D-3L and EORTC QLQ-C30 showed a significantly higher cumulative incidence of a favorable HR-QoL outcome than an unfavorable one; however, no significant difference was observed using the SF-36. The EQ-5D is a widely used generic instrument that provides a simple, preference-based measure of health status across 5 domains: mobility, self-care, usual activities, pain/discomfort, and anxiety/depression.^11^ In contrast, the EORTC QLQ-C30 is a cancer-specific, non-preference-based tool developed for oncology populations. It offers a detailed assessment of symptoms and functional domains relevant to cancer patients, but it does not yield utility scores for economic modeling.^12^ The SF-36, another generic measure, assesses a broad range of health concepts spanning 8 health domains. Like the EORTC QLQ-C30, it is not preference-based.^13^ It is unclear whether this might account for the differences in metrics we observed, as there have been no previous published studies that compare 2 or more HR-QoL instruments in a cohort of diffuse glioma patients. We also note that SF-36 data in our pooled analysis came from a single study that included separate cohorts of LGG and HGG patients, with the physical component and mental component scores reported separately.^29^

In other forms of cancer, comparative studies of the non-preference-based SF-36 and EORTC QLQ-C30 instruments have shown that they perform similarly. In a cross-sectional study of 604 patients with breast and colon cancer, good correlations were observed between the SF-36 and EORTC QLQ-C30 across several domains, particularly in physical and psycho-social scales.^34^ In another study using the International Classification of Functioning, Disability and Health system to identify commonalities among a variety of HR-QoL instruments in patients with cancer, the physical function domains of the EORTC QLQ-C30 and SF-36 were found to be more similar than the FACT-G instrument.^35^

In contrast, attempts to compare HR-QoL trends in the preference-based EQ-5D with the SF-36 or EORTC QLQ-C30 tend to be more challenging. When comparing the performance of the EQ-5D-5L and QLQ-C30 in breast cancer patients, Vrancken Peeters et al.^36^ observed a higher degree of internal responsiveness of the EQ-5D-5L in detecting changes in health status, particularly within the first 6 months following surgery; however, the changes were below the MCID. In our study, improvements in HR-QoL as measured by the EQ-5D were more apparent and significant at earlier time points than those measured by either the EORTC QLQ-C30 or SF-36, which could be due to a combination of greater internal responsiveness in the glioma population and the substantially larger size of the EQ-5D subgroup compared to the other 2, increasing its power to detect HR-QoL change. In attempting to address these challenges, Yousefi et al.^37^ performed a mapping of the SF-36 into a preference-based tool, the SF-6D, by applying valuation coefficients to SF-36 items using the standard gamble method. However, disparities in these instruments remained, with EQ-5D utility scores skewing toward higher values, resulting in a ceiling effect and limited ability to discriminate between higher-valued health states compared to the SF-6D. Although SF-6D scores were more normally distributed, a relatively high minimum bound was observed, contributing to poor agreement with the EQ-5D at the lower end of the health utility scale as well. Future longitudinal HR-QoL studies in diffuse glioma patients would benefit from the complementary use of preference-based and non-preference-based instruments. The decision to use cancer-specific versus generic instruments should also be guided by careful consideration of how best to understand the study participant’s lived experience and capture the impact of the clinical changes most relevant to their condition.

Unsurprisingly, our meta-regression analysis indicated that studies with a larger proportion of patients with HGG were also more likely to report worse HR-QoL compared to their preoperative baseline at time points later than 3 months after surgery. This is consistent with the notion that high-grade diffuse gliomas progress more rapidly than low-grade diffuse gliomas, leading to a more rapid development of neurological deficits, functional decline, and consequently deteriorating HR-QoL. Coomans et al.^33^ found that patients with GBM declined more rapidly than patients with grade 2 or 3 gliomas on HR-QoL symptom domains, including fatigue, drowsiness, and motor dysfunction. Other studies, including both LGG and HGG patients generally, show that HGG patients report lower HR-QoL than LGG patients, but because of their cross-sectional nature, they lack the longitudinal assessments necessary to make conclusions about relative rates of deterioration and disease progression.^38–40^

Interestingly, our meta-regression analysis indicated that studies with younger patients, while controlling for the proportion of HGG patients, were more likely to report worsened HR-QoL, both within and after 3 months of undergoing resection. Although it is established that lower age is predictive of improved survival in patients with both low-^41^^,^^42^ and high-grade diffuse gliomas,^43^^,^^44^ our findings suggest that changes in HR-QoL and survival do not necessarily parallel each other. In a prospective cohort study involving 309 patients with diffuse gliomas of varying ages, Renovanz et al.^45^ observed that levels of psychological distress and emotional functioning were similar in both younger and older patients, but older patients scored lower on the physical and cognitive function scales of QLQ-C30 and QLQ-BN20. Thus, the detrimental impact of surgically induced motor and cognitive deficits on HR-QoL may be more apparent in younger patients than elderly patients. It was unclear, however, how much of the HR-QoL data came from patients prior to surgical intervention, emphasizing the need for future studies to clarify the trends in HR-QoL score differences between younger and older patients.

Studies with a higher GTR rate tended to report worse HR-QoL outcomes within the first 3 months after surgery, but this trend reversed at later time points, wherein a higher GTR rate was independently predictive of improved HR-QoL. In a trial of 322 GBM patients, fluorescence-guided surgery was found to improve rates of GTR but was also associated with a higher rate of new functional deficits compared to patients undergoing standard resection (24% vs. 15%).^46^ This difference, however, was only apparent at 48 h postoperatively and was not seen at 1 or 6 weeks postoperatively. Another retrospective study by Gulati et al.^47^ and a more recent randomized trial on fluorescence-guided surgery,^48^ found no association between GTR rate and neurological deficits up to 3 months postoperatively. We were unable to identify any studies that directly compared HR-QoL outcomes among groups of diffuse glioma patients that differed by EOR. Thus, while surgically induced neurological deficits may provide a convincing explanation for transient HR-QoL worsening, strong evidence for this in the literature is currently lacking, meriting further exploration.

Because the EOR threshold for an overall survival benefit has been thought of as higher for HGGs (>80%)^47^^,^^49^ than for LGGs (>70%),^48^^,^^50^ we also examined for a correlation between %HGG and %GTR among our studies, but found none. Our analysis was limited by inconsistent EOR reporting methods among the studies and frequently missing tumor eloquence grading data, which is a potential confounding variable. In future studies, more consistent use of EOR reporting standards and measures of tumor resectability will facilitate meta-analyses that better elucidate the relationship between EOR, tumor grade, and HR-QoL.

Our findings will aid clinicians in identifying and intervening in patients at risk of HR-QoL deterioration as early as possible during oncologic treatment. The highest risk subgroup may be younger patients with HGGs undergoing aggressive resections near eloquent brain regions, since surgeons may understandably prioritize EOR and survival time at the expense of inducing temporary neurological deficits. Randazzo and Peters^51^ highlighted the increased risk of HR-QoL deterioration in younger patients with primary brain tumors, particularly due to stress regarding family and employment concerns. For these patients, clinicians may need to focus on addressing modifiable psychosocial factors that influence HR-QoL after surgery. Wassef et al.^52^ identified factors such as resilience, spirituality, and end-of-life concerns to be of importance and suggested several evidence-based interventions for HR-QoL improvement, including neurocognitive rehabilitation, sleep and exercise hygiene, and caregiver training.

### Limitations

This study is limited by the relatively low number of studies included, which reduces its power to detect predictors of HR-QoL trends we observed. We included 4 studies conducted by the same author/institution, published within a 6-year period. While each study reported different samples, it is possible that patients overlapped. Data for the SF-36 subgroup analysis came from a single multi-cohort study conducted at a single institution. Since the MCID for the SF-36 varies by patient population, the trend toward worsening HR-QoL at T1 in this subgroup might have limited generalizability. Heterogeneity among the studies in protocols for postoperative counseling and caretaker support could have been a confounding variable in our meta-regression analyses. Additionally, some of the improvements in HR-QoL observed at later timepoints may reflect patient adaptation to a new health state, rather than any effect of treatment, but there was insufficient patient-level data on disease progression to determine this with certainty. Finally, there were inconsistencies in the thresholds used for the MCID among the 3 studies using the EORTC-QLC C30, which might reduce the validity of our pooled odds and cumulative incidence results.

## Conclusion

Surgical resection in patients with diffuse gliomas is associated with a shift toward stable or improved HR-QoL outcomes over time, particularly beyond the initial 3 months after surgery. Younger age, higher glioma grade, and lower rates of GTR were predictors of worsened long-term HR-QoL outcomes, highlighting opportunities for the early identification of at-risk patients for targeted interventions. In subgroup analyses, differences between the EQ-5D-3L, EORTC QLQ-C30, and SF-36 underscore the complexity of comparing preference-based, non-preference-based, generic, and cancer-specific measures and highlight the value of using complementary instruments to capture the full trajectory of HR-QoL in glioma patients. Future studies in diffuse glioma patients should exercise care in the selection and interpretation of HR-QoL metrics to enhance comparability and data synthesis.

## Supplementary Material

npaf111_Supplementary_Data

## Data Availability

All data generated and analyzed in this study are derived from the studies listed in Table 1 and are available by request from the corresponding author.
