# Supplementary material for: Health-related quality of life outcomes of surgery for diffuse glioma: A systematic review and pooled analysis
Source: Neurooncol Pract. 2025 Oct 25;13(1):60–70. doi: 10.1093/nop/npaf111 (PMC12965647; doi:10.1093/nop/npaf111)
Supplement: npaf111_Supplementary_Data [file npaf111_supplementary_data.zip › Supplementary Table Final (20250918).docx]

Supplementary Table 1. Study Bias Assessment

| Newcastle Ottawa Scale (Prospective Cohort Studies) | | | | | | | | | | | | | | | | |
| --- | --- | --- | --- | --- | --- | --- | --- | --- | --- | --- | --- | --- | --- | --- | --- | --- |
| *Study* | *Representativeness of Exposed Cohort* | *Selection of Non-exposed Cohort* | *Ascertainment of Exposure* | | *Demonstrating that outcome of interest was not present at start of study* | | *Comparability of Cohorts on the basis of design/ analysis* | | *Assessment of Outcome* | | *Long-enough Follow-up* | | *Adequacy of follow-up of Cohorts* | | *Total* | |
| Jakola, Gulati et al. (2011)^20^ | 1 | 0 | 1 | | 1 | | 1 | | 1 | | 1 | | 1 | | 7 | |
| Jakola et al. (2011)^21^ | 1 | 0 | 1 | | 1 | | 1 | | 1 | | 1 | | 0 | | 6 | |
| Jakola et al. (2015)^22^ | 1 | 0 | 1 | | 1 | | 1 | | 1 | | 1 | | 0 | | 6 | |
| Sadberg et al. (2016)^23^ | 1 | 0 | 1 | | 1 | | 1 | | 1 | | 1 | | 1 | | 7 | |
| Wolf et al. (2016)^24^ | 1 | 0 | 1 | | 1 | | 1 | | 1 | | 1 | | 0 | | 6 | |
| Jakola et al. (2017)^25^ | 1 | 0 | 1 | | 1 | | 1 | | 1 | | 1 | | 0 | | 6 | |
| Drewes et al. (2018)^26^ | 1 | 1 | 1 | | 1 | | 1 | | 1 | | 1 | | 0 | | 7 | |
| Sadberg et al. (2019)^27^ | 1 | 0 | 1 | | 1 | | 1 | | 1 | | 1 | | 0 | | 6 | |
| Leonetti et al. (2021)^29^ | 1 | 1 | 1 | | 1 | | 1 | | 1 | | 1 | | 0 | | 7 | |
| Taskiran et al. (2021)^30^ | 1 | 0 | 1 | | 1 | | 1 | | 1 | | 1 | | 1 | | 7 | |
| Rubin et al. (2022)^31^ | 1 | 1 | 1 | | 1 | | 1 | | 1 | | 1 | | 0 | | 7 | |
| Cochrane Risk of Bias 2.0 Tool (Clinical Trials) | | | | | | | | | | | | | | | | |
|  | Random Allocation | Concealed Allocation | | Blinding | | Similar Care | | Incomplete Outcomes | | Free of Suggestion | | Free of Other Problems | | - | | - |
| Liu et al. (2022)^28^ | Low Bias | Low Bias | | Unclear | | Low Bias | | High Bias | | Low Bias | | Low Bias | | - | | - |
